# Predictors for Mortality among Multidrug-Resistant Tuberculosis Patients in Tanzania

**DOI:** 10.1155/2017/9241238

**Published:** 2017-07-20

**Authors:** Edson W. Mollel, Jaffu O. Chilongola

**Affiliations:** ^1^Kibong'oto Infectious Diseases Hospital, P.O. Box 12, Sanya Juu, Siha, Tanzania; ^2^Kilimanjaro Christian Medical University College, P.O. Box 2240, Moshi, Tanzania; ^3^Kilimanjaro Clinical Research Institute, P.O. Box 2236, Moshi, Tanzania

## Abstract

**Problem:**

Factors related to MDRTB mortality in Tanzania have not been adequately explored and reported.

**Objectives:**

To determine demographic, clinical, radiographic, and laboratory factors associated with MDRTB mortality in a Tanzanian TB Referral Hospital.

**Methodology:**

This was a cross-sectional study with 193 participants. Demographic, clinical, laboratory, and radiological data were collected, and their associations with mortality among MDRTB patients were determined.

**Results and Conclusions:**

Cough was the commonest finding among these MDRTB patients, with 179 (92.75%) of them presenting with cough, followed by chest X-ray consolidation in 156 patients (80.83%) and history of previous TB treatment in 151 patients (78.24%). Cigarette smoking, HIV positivity, and low CD4 counts were significantly associated with MDRTB mortality, *p* values of 0.034, 0.044, and 0.048, respectively. Fever on the other hand was at the borderline with *p* value of 0.059. We conclude that cigarette smoking and HIV status are significant risk factors for mortality among MDRTB patients. HIV screening should continually be emphasized among patients and the general community for early ARTs initiation. Based on the results from our study, policy makers and public health personnel should consider addressing tobacco cessation as part of national TB control strategy.

## 1. Introduction

Multidrug-resistant tuberculosis (MDRTB), defined as resistance to both isoniazid and rifampicin, is a growing public health problem in resource-poor regions where adequate diagnosis and treatment are often unavailable. The WHO estimated 450,000 new cases of MDRTB in 2012 [1, 2]. It is a growing public health concern, with an estimation of 3.5% of new TB cases and 20.5% for those previously treated for TB to turning to MDRTB. WHO estimates that about 5% of all TB cases progress to MDRTB, of which more than 40% died in 2013 [3, 4]. Treatment of MDR- and XDR-TB is difficult even in resource-rich settings. Patients are generally treated for a minimum of 18–24 months with second-line TB drugs that have significant adverse effects [3]. There is now a new regimen for treating new and uncomplicated cases of MDRTB for less than one year.

MDRTB and extensively drug-resistant tuberculosis (XDR-TB) are severe global public health threats [3, 5, 6]. At least one country in every region of the world has reported a high prevalence of MDRTB (more than 3% of all new TB cases), and XDR-TB has been diagnosed in more than 57 countries [5]. Recent reports suggest that the prevalence of drug-resistant TB in sub-Saharan Africa may be increasing [7–9], raising concerns for a disastrous convergence with the continent's generalized HIV/AIDS epidemic. While global data show that only 48% of the MDRTB patients are successfully treated largely due to high mortality and loss to follow-up, in Tanzania about 1.1% (0.5%–2%) and 3.1% (0.9%–7.9%) of new and retreatment TB cases are MDRTB. Of the MDRTB patients in Tanzania, it is estimated that 75% of them start treatment [4]. A previous study done at the study site of Kibong'oto Hospital on MDRTB patients found that about 7% of MDRTB patients died, 5% defaulted, and 89% completed the intensive phase [10]. Therefore, death and defaulting together made a total of more than 10% of all MDRTB patients just after finishing intensive phase of treatment.

Previous studies have determined the factors associated with MDRTB mortality, including immunosuppression, degree of resistance to antituberculosis drugs [11], malnutrition, defaulting, or loss to follow-up [12]. Some of these factors may be embedded with social cultural characteristics of patients such as behavior and lifestyle and sociodemographic characteristics. Only one report has been published in Tanzania to assess risks for mortality among MDRTB patients. A study by Mpagama and colleagues established delay in diagnosis and treatment initiation among MDRTB patients to be an important risk for mortality [10]. Despite the rapidly changing epidemiology of MDRTB, HIV, and TB-HIV coinfection rates and lifestyles in the society, there is no current data on factors associated with MDRTB mortality in Tanzania. We have designed the current study to determine associations that exist between demographic, clinical, radiographical, and laboratory factors on one hand and MDRTB mortality on the other hand. This information is important for clinicians in sharpening their suspicion index on MDRTB poor prognostic factors at diagnosis and enabling timely refocusing of treatment regimens, prevention, and control strategies against MDRTB.

## 2. Materials and Methods 

### 2.1. Study Site and Design

This was a retrospective file review using patient files as source of data. The study involved files of patients older than 15 years of age, admitted at Kibong'oto Infectious Diseases Hospital (KIDH) MDRTB wards from 1 January 2012 to 30 of June 2014. KIDH is the country's national referral center for treatment of the MDRTB cases. It is located in Kilimanjaro Region, 15 kilometers from the highway connecting Moshi and Arusha towns. It has around 340 beds for tuberculosis patients, and around 100 patients are seen daily as outpatients. MDRTB treatment started in 2009 at KIDH, when it was the only center for MDRTB treatment. Efforts have been done to decentralize the management of these patients. Currently, patients would start MDRTB treatment at their nearest health facility. Initially, all MDRTB patients would have to come and stay at KIDH for intensive phase of treatment which was defined as the time from starting MDRTB treatment to 2 months after sputum culture conversion (this duration was later standardized to 8 months for all patients). The following regimen is used for MDRTB treatment: Kanamycin or Capreomycin, Levofloxacin, Cycloserine, Ethionamide, Pyrazinamide, and Ethambutol. These patients are being monitored by monthly sputum smear and culture.

### 2.2. Sampling and Study Procedures

Patient files of MDRTB cases admitted at KIDH in the period from 1 January 2012 to 30 June 2014 were reviewed. Files of patients below 15 years and those with missing clinical data for the study were excluded. Patients' independent and dependent variables were collected and entered into an Excel sheet. The following were the study's independent variables: demographic parameters, which included age, sex, domicile, education, occupation, cigarette smoking history, history of alcohol intake, and mining. Clinical parameters such as cough, chest pain, fever, weight loss, night sweat, haemoptysis, easy fatigability, shortness of breath, previous TB treatment, HIV status, and BMI were recorded. Hematological and biochemistry parameters recorded included hemoglobin, alanine aminotransferase, aspartate aminotransferase, and CD4+ count. Presence/absence of consolidation, cavities, pleural effusion, and lymphadenopathy in chest X-ray were collected as radiological parameters. Mortality of any cause during MDRTB treatment was considered as the dependent variable.

### 2.3. Data Analysis

Patients who did not meet the inclusion criteria were excluded so as to clean the data. Data were then transferred from the Excel spread sheet into STATA v14.1 for analysis. Pearson chi-square test was used to determine the association between mortality and categorical independent factors. Fisher's exact test was used where the data was less than 5 in any single cell. For normally distributed data and skewed data, Student's *t*-test and Wilcoxon rank-sum tests were used respectively.

### 2.4. Ethics

Permission from KIDH authority to conduct the study was sought. Patients' privacy and confidentiality were strictly observed throughout the study. Ethical clearance was obtained from KCMUCo's College Research and Ethical Review Committee (CRERC).

## 3. Results

Demographic information of participants is presented in Table 1. In total, 193 patients' files were reviewed. The median and mean ages were 38 years and 39.5 years, respectively, with 67.32% of them being males. Out of the 193 patients whose files were reviewed in this study, 180 (93.26%) were alive while 13 patients (6.74%) died (Table 1). Sixty-one patients (41.61%) and 76 (51.70%) of all patients who were alive had history of smoking and alcohol intake, respectively.

Table 2 summarizes results for demographical predictors for MDRTB mortality. Cigarette smoking and being HIV positive were significant predictors for mortality (OR, 5.44, 95% CI: 1.09–27.19, *p* = 0.039; OR 3.4595% CI: 1.022–11.64, *p* = 0.046), respectively. Eighty of all patients were HIV positive, and none of these MDRTB patients had unknown HIV status. Alcohol drinking and sex were not predictors of MDRTB mortality.

Table 3 shows that none of the clinical predictors was associated with MDRTB mortality. However, despite not reaching statistical significance, fever was the only clinical predictor closely associated with mortality (*p* = 0.059). Cough was the most common clinical predictor among MDRTB patients. Eleven patients (85%) of those who died and 140 (78%) of those who were alive presented with more than one episode of TB treatment. Nine patients (100%) of those who died and 91 (69%) of those who were alive had fever. One hundred and fifty-five of the patients (80.31%), 129 patients (66.84%), and 100 patients (51.81%) had missing information with regard to fatigability, dyspnea, and weight loss, respectively.

Regarding hematological and biochemistry parameters (Table 4), only low CD4+ count (<200 cells/ul) was found to predict mortality for MDRTB (OR 0.99,95% CI = 0.98–0.999, *p* = 0.048). None of the radiographic parameters studied predicted MDRTB mortality (Table 5). Chest X-ray consolidation was the most common radiological findings. Only nine patients, presented with pleural effusion in this study.

## 4. Discussion

The objective of this study was to determine the factors associated with MDRTB mortality at KIDH settings. We have found that cigarette smoking was related to MDRTB mortality. Our findings are in agreement with other studies elsewhere [13, 14]. Cigarette smoking is speculated to lower cytokine-producing macrophages with diminished influx of interferon gamma producing effector T-cells in the lungs. This prevents the filtering mechanism system that inhibits bacteria from reaching the lungs which, in turn, leads to increased incidences of active and latent pulmonary TB as well as poor clinical prognosis [15]. Smoking has been associated with failure to achieve a suppressed HIV viral load in a study of HIV patients [16]. Further, tobacco smoking has been shown to significantly increase oxidative stress among smoking, in HIV-infected individuals [17]. Apart from worsening TB through interfering with cytokine processes in the lungs, cigarette smoking could also affect MDRTB prognosis through the later two mechanisms [13, 14], meaning that cigarette smoking could affect MDRTB prognosis indirectly through manipulating other factors including suppression of anti-TB drug activity.

The consequences of tobacco on worsening active TB morbidity and mortality are augmented not only by active smoking but also by passive smoke, second-hand smoke, and environmental tobacco smoke exposure [18]. Smokers tend to have a late TB diagnosis as symptoms tend to be mistaken for smoking effects [19], leading to late initiation of treatment which result in poor outcomes. Scientific evidence shows that even when treated for TB, relapse risk is also higher for MDRTB patients with history of smoking [18]. Other studies have shown a link between cigarette smoking and increased risk of transmission to child contacts [20, 21]. Collectively, the effect of cigarette smoking on poor MDRTB prognosis is unarguably confirmed.

Our data show an association between HIV positivity and MDRTB mortality in way that HIV positive MDRTB patients had higher mortality rates than those HIV negative patients. This observation is not different from previous studies that obtained similar findings [22–24]. With reduced integrity and function of the CD4+ cells, HIV-infected patients are at an increased risk of acquiring TB even when on ARTs [25, 26] and an increased risk of progressing at a rapid rate to an active TB [27] and at the greatest proportion compared to HIV-uninfected patients. TB on the other hand increases HIV replication and viral diversification rates, by increasing proinflammatory cytokine production which increases HIV viral replication and diversity and so facilitating immune escape [28–31]. This synergistic effect could be the principal reason for the high mortality among HIV coinfected MDRTB patients. But on the other hand, being a male on ARTs prior to initiation of the MDRTB treatment was associated with high cure rate among HIV coinfected MDRTB patients [32]. Therefore, regardless of HIV increased risk of mortality, patients with earlier detection of HIV and earlier initiation of ARTs are more likely to have a good treatment outcome. Hence, HIV screening program and active initiation of ARTs should be strongly advocated.

In the current study, lower CD4+ counts were associated with higher MDRTB mortality. Lower CD4+ counts are indicative of reduced immunity due to HIV infection progression. We have reported in the current study an association between HIV positivity and MDRTB mortality. CD4+ count is a cardinal marker for HIV infection progression and thus the degree of immune suppression. The observed association between low CD4+ (<200) counts and higher MDRTB mortality can be explained as an indirect immunosuppressive effect of HIV infection. This association has long been established by previous studies [6, 11, 33–35]. Efforts to reduce mortality due to MDRTB must, among other strategies, focus on an aggressive HIV testing and ART initiation.

None of the remaining parameters, clinical, lab, and radiological factors, were significantly associated with MDRTB mortality. Other studies have found other factors to be related to MDRTB mortality, such as immunosuppression and drug resistance patterns [11], lower educational levels, previous TB episodes and diabetes history [22], other complications [13], anemia, positive sputum smear, hepatitis, drug use, diabetes, and history of previous TB [14], which were not part of our study. Our study had the inherent limitation of being a retrospective study, with several missing data entries in the data base, and thus a smaller sample size for the analyses could have affected statistical power of associations.

## 5. Conclusions

In conclusion, cigarette smoking, HIV positivity, and low CD4 count are significant risk factors for higher mortality rates among MDRTB patients. We recommend that HIV screening be emphasized among TB patients and the general community for early ARTs initiation. Based on the results from our study, policy makers and public health personnel should consider addressing tobacco cessation as part of national TB control strategy.

## Figures and Tables

**Table 1:** Descriptive sociodemographic characteristics of study participants (*N* = 193).

Characteristics	*n* (% of total)
*Age*	
11–20 years	11 (5.70)
21–50 years	146 (75.65)
>50 years	36 (18.65)
*Sex*	
Female	63 (32.64)
Male	130 (67.36)
*Occupation*	
Business	78 (41.27)
Employed	19 (10.05)
Farmer	70 (37.04)
House wife	6 (3.17)
Miner	3 (1.59)
Student	13 (6.88)
*Alcohol*	
No	71 (48.30)
Yes	76 (51.70)
*Cigarette smoking*	
No	86 (58.50)
Yes	61 (41.50)
*HIV status*	
Negative results	113 (58.55)
Positive results	80 (41.45)
*Mortality*	
Alive	180 (93.26)
Died	13 (6.74)
*Comorbidity* ^*∗*^	
No	113 (58.55)
Yes	80 (41.45)

^*∗*^Comorbidity = MDR-TB + HIV.

**Table 2:** Demographic data for predictors of MDRTB mortality.

Baseline demographic predictors	Died (*n* = 13)	Alive (*n* = 180)	OR (95% CI)	*p* value (chi-square test)
*Age (n = 193)*				
Mean ± SD	44 ± 5.6	39 ± 0.9	—	0.193^*∗*^
Median (IQR)	38 (34–54)	38 (31–47.5)		
*Sex (n = 193)*				
Male	10 (77%)	120 (67%)	1.67 (.44–6.28)	0.45
Female	3 (23%)	60 (33%)		
*Alcohol (n = 147)*				
Yes	4 (57%)	72 (51%)	1.26 (0.27–5.83)	0.768
No	3 (43%)	68 (49%)		
*Cigarette smoking (n = 147)*				
Yes	7 (78%)	54 (39%)	5.44 (1.09–27.19)	0.039
No	2 (22%)	84 (61%)		
*HIV (n = 193)*				
Yes	9 (69%)	71 (39%)	3.45 (1.02–11.64)	0.046
No	4 (31%)	109 (61%)		

^*∗*^ *t*-test was done for continuous variables.

**Table 3:** Clinical predictors of MDRTB mortality.

Baseline clinical predictors	Died (*n* = 13)	Alive (*n* = 180)	OR (95% CI)	*p* values (chi-square test)
*Cough (n = 182)*				
Yes	11 (100%)	168 (98%)	—	0.999
No	0 (0%)	3 (2%)		
*Chest pain (n = 182)*				
Yes	9 (100%)	98 (88%)	—	0.595
No	0 (0%)	13 (12%)		
*Fever (n = 141)*				
Yes	9 (100%)	91 (69%)	—	0.059
No	0 (0%)	41 (31%)		
*Weight loss (n = 93)*				
Yes	3 (100%)	84 (93%)	—	0.999
No	0 (0%)	6 (7%)		
*Night sweat (n = 116)*				
Yes	4 (100%)	86 (77%)	—	0.573
No	0 (0%)	26 (23%)		
*Hemoptysis (n = 73)*				
Yes	4 (80%)	36 (53%)	3.56 (0.38–33.48)	0.268
No	1 (20%)	32 (47%)		
*Easily fatigability (n = 38)*				
Yes	5 (100%)	29 (88%)	—	0.999
No	0 (0%)	4 (12%)		
*Dyspnea (n = 64)*				
Yes	3 (100%)	53 (87%)	—	0.999
No	0 (0%)	8 (13%)		
*Previous TB treatment (n = 192)*				
Yes	11 (85%)	140 (78%)	1.53 (0.33–7.20)	0.589
No	2 (15%)	39 (22%)		
*Weight (n = 193)*				
Mean ± SD	50.3 ± 2.8	51.3 ± 0.8	—	0.749^*∗*^
*BMI (n = 131)*				
Mean ± SD	19.3 ± 0.8	18.7 ± 0.3	—	0.624^*∗*^

^*∗*^ *t*-test was done for continuous variables.

**Table 4:** Hematological and clinical biochemistry predictors of MDRTB mortality.

Baseline lab predictors	Died (*n* = 13)	Alive (*n* = 180)	*p* values^*∗*^
*Hemoglobin (n = 151)*			
Mean ± SD	10 ± 0.8	11.4 ± 0.2	0.135
*Alanine aminotransferase (n = 131)*			
Mean ± SD	22.5 ± 3.2	20 ± 1.7	0.71
*Aspartate aminotransferase (n = 128)*			
Mean ± SD	33.88 ± 7.1	29.8 ± 1.4	0.486
*CD4 count (n = 70)*			
Mean ± SD	77 ± 30	316 ± 33	0.048

SD: standard deviation; ^*∗*^*t*-test was done for continuous variables.

**Table 5:** Radiological predictors of MDRTB mortality.

Baseline radiological predictor	Died (*n* = 13)	Alive (*n* = 180)	OR (95% CI)	*p* value (chi-square test)
*Consolidation (n = 170)*				
Yes	12 (100%)	144 (91%)	—	0.602
No	0 (0%)	14 (9%)		
*Cavities (n = 170)*				
Yes	1 (8%)	48 (30%)	0.2 (0.03–1.66)	0.138
No	11 (92%)	110 (70%)		
*Pleural effusion (n = 170)*				
Yes	0 (0%)	9 (6%)	—	0.999
No	12 (100%)	149 (94%)		
*Lymphadenopathy (n = 170)*				
Yes	7 (58%)	62 (39%)	2.17 (0.66–7.13)	0.203
No	5 (42%)	9 (661%)		

## References

[1] Zumla A., George A., Sharma V., Herbert R. H. N., Oxley A., Oliver M. (2015). The WHO 2014 global tuberculosis report—further to go. *The Lancet Global Health*.

[2] WHO (2013). *Global tuberculosis report 2013. World Health Organization*.

[3] Mukherjee J. S., Rich M. L., Socci A. R. (2004). Programmes and principles in treatment of multidrug-resistant tuberculosis. *The Lancet*.

[4] World Health Organization. Antimicrobial resistance: 2014 global report on surveillance. World Health Organization; 2014

[5] World Health Organization. Global tuberculosis control: WHO report 2010. World Health Organization; 2010

[6] Gandhi N. R., Shah N. S., Andrews J. R. (2010). HIV coinfection in multidrug- and extensively drug-resistant tuberculosis results in high early mortality. *American Journal of Respiratory and Critical Care Medicine*.

[7] Amor Y. B., Nemser B., Singh A., Sankin A., Schluger N. (2008). Underreported threat of multidrug-resistant tuberculosis in Africa. *Emerging Infectious Diseases*.

[8] Buthelezi S. S. S. Situational analysis of TB drug resistance in KwaZulu-Natal province: Republic of South Africa.

[9] Chirenda J., Menzies H., Moalosi G., Anisimova V., Radisowa K., Bachhuber M. (2009). *The trend of resistance to anti-tuberculosis drugs in Botswana: results from the 4th national anti-tuberculosis drug resistance survey*.

[10] Mpagama S. G., Heysell S. K., Ndusilo N. D. (2013). Diagnosis and interim treatment outcomes from the first cohort of multidrug-resistant tuberculosis patients in Tanzania. *PLoS ONE*.

[11] Gandhi N. R., Andrews J. R., Brust J. C. M. (2012). Risk factors for mortality among MDR- and XDR-TB patients in a high HIV prevalence setting. *International Journal of Tuberculosis and Lung Disease*.

[12] Brust J. C. M., Gandhi N. R., Carrara H., Osburn G., Padayatchi N. (2010). High treatment failure and default rates for patients with multidrug-resistant tuberculosis in KwaZulu-Natal, South Africa, 2000-2003. *International Journal of Tuberculosis and Lung Disease*.

[13] Molalign S., Wencheko E. (2016). Risk factors of mortality in patients with multi-drug resistant TB. *The Ethiopian Journal of Health Development*.

[14] Alavi-Naini R., Moghtaderi A., Metanat M., Mohammadi M., Zabetian M. (2013). Factors associated with mortality in tuberculosis patients. *Journal of Research in Medical Sciences*.

[15] Bonacci R. A., Cruz-Hervert L. P., García-García L. (2013). Impact of cigarette smoking on rates and clinical prognosis of pulmonary tuberculosis in Southern Mexico. *Journal of Infection*.

[16] Mdodo R., Frazier E. L., Dube S. R. (2015). Cigarette smoking prevalence among adults with HIV compared with the general adult population in the United States: cross-sectional surveys. *Annals of Internal Medicine*.

[17] Ande A., McArthur C., Ayuk L. (2015). Effect of mild-to-moderate smoking on viral load, cytokines, oxidative stress, and cytochrome P450 enzymes in HIV-infected individuals. *PLoS ONE*.

[18] Wang J., Shen H. (2009). Review of cigarette smoking and tuberculosis in China: intervention is needed for smoking cessation among tuberculosis patients. *BMC Public Health*.

[19] Wen C.-P., Chan T.-C., Chan H.-T., Tsai M.-K., Cheng T.-Y., Tsai S.-P. (2010). The reduction of tuberculosis risks by smoking cessation. *BMC Infectious Diseases*.

[20] Lönnroth K., Jaramillo E., Williams B. G., Dye C., Raviglione M. (2009). Drivers of tuberculosis epidemics: the role of risk factors and social determinants. *Social Science & Medicine*.

[21] Huang C.-C., Tchetgen E., Becerra M. C. (2014). Cigarette smoking among tuberculosis patients increases risk of transmission to child contacts. *International Journal of Tuberculosis and Lung Disease*.

[22] Chung-Delgado K., Guillen-Bravo S., Revilla-Montag A., Bernabe-Ortiz A. (2015). Mortality among MDR-TB cases: comparison with drug-susceptible tuberculosis and associated factors. *PLoS ONE*.

[23] Yema A. (2016). *Survival and Its Determinant of Multidrug-Resistant Tuberculosis Patients with HIV Co-Infection*.

[24] Manda SO., Masenyetse LJ., Lancaster JL., van der Walt ML. (2013). Risk of death among HIV co-infected multidrug resistant tuberculosis patients, compared to mortality in the general population of South Africa. *Journal of AIDS clinical research*.

[25] Gupta A., Wood R., Kaplan R., Bekker L.-G., Lawn S. D. (2012). Tuberculosis incidence rates during 8 years of follow-up of an antiretroviral treatment cohort in south africa: Comparison with rates in the community. *PLoS ONE*.

[26] Sonnenberg P., Murray J., Glynn J. R., Shearer S., Kambashi B., Godfrey-Faussett P. (2001). HIV-1 and recurrence, relapse, and reinfection of tuberculosis after cure: a cohort study in South African mineworkers. *Lancet*.

[27] Holmes C. B., Wood R., Badri M. (2006). CD4 decline and incidence of opportunistic infections in Cape Town, South Africa: Implications for prophylaxis and treatment. *Journal of Acquired Immune Deficiency Syndromes*.

[28] Kalsdorf B., Skolimowska K. H., Scriba T. J. (2013). Relationship between chemokine receptor expression, chemokine levels and HIV-1 replication in the lungs of persons exposed to Mycobacterium tuberculosis. *European Journal of Immunology*.

[29] Juffermans N. P., Speelman P., Verbon A. (2001). Patients with active tuberculosis have increased expression of HIV coreceptors CXCR4 and CCR5 on CD4+ T cells. *Clinical Infectious Diseases*.

[30] Kinoshita S., Chen B. K., Kaneshima H., Nolan G. P. (1998). Host control of HIV-1 parasitism in T cells by the nuclear factor of activated T cells. *Cell*.

[31] Toossi Z., Johnson J. L., Kanost R. A. (2001). Increased replication of HIV-1 at sites of Mycobacterium tuberculosis infection: potential mechanisms of viral activation. *Journal of Acquired Immune Deficiency Syndromes*.

[32] Umanah T., Ncayiyana J., Padanilam X., Nyasulu P. S. (2015). Treatment outcomes in multidrug resistant tuberculosis-human immunodeficiency virus Co-infected patients on anti-retroviral therapy at Sizwe Tropical Disease Hospital Johannesburg, South Africa. *BMC Infectious Diseases*.

[33] Martin D. J., Sim J. G., Sole G. J. (1995). CD4+ lymphocyte count in African patients co-infected with HIV and tuberculosis. *Journal of Acquired Immune Deficiency Syndromes & Human Retrovirology*.

[34] McDyer J. F., Hackley M. N., Walsh T. E., Cook J. L., Seder R. A. (1997). Patients with Multidrug-Resistant Tuberculosis with Low CD4+ T Cell Counts Have Impaired Th1 Responses. *Journal of Immunology*.

[35] Salomon N., Perlman D. C., Friedmann P., Buchstein S., Kreiswirth B. N., Mildvan D. (1995). Predictors and outcome of multidrug-resistant tuberculosis. *Clinical Infectious Diseases*.

